# The support of Pelé: how to spread the compliance and commitment to hand hygiene

**DOI:** 10.1186/2047-2994-4-S1-P154

**Published:** 2015-06-16

**Authors:** HIG Giamberardino, JA Carneiro

**Affiliations:** 1 Epidemiology and Infection Control Department, Hospital Pequeno Principe, Curitiba, Brazil; 2 Hospital Pequeno Principe, Curitiba, Brazil

## Introduction

The Little Prince Hospital (LPH), since 1919 is the largest paediatric teaching hospital in Brazil, maintaining an environment for innovation together with deep compromise with the patients assisted. The LPH is a philanthropic and no-profit organization with the support of brazilian celebrities, such as our most famous volunteer Edson Arantes do Nascimento, worldwide known as Pelé, who gave his name to our research institute (since 2005). Pelé brings us opportunities related to visibility and fundraising. We are currently celebrating 10 years of partnerships with Pelé, serving the cause of children health and engaging himself in spreading good practices in healthcare.

## Objectives

To support efforts and improve staff compliance with hand hygiene practices according to the World Health Organization (WHO) Global Campaign: SAVE LIVES: Clean Your Hands.

## Methods

Pelé accepted our invitation to learn the handrubbing technic according to the WHO “How to Handrub” methodology and poster. Then, he agreed photos to be taken while he was rubbing his hands and for a video to be produced in the support of infection prevention initiatives.

## Results

Create educative materials like videos can be broadcasted both internally and worldwide, with the support of local health authorities and national and international stakeholders. Whereas the impact of such a strategy is still unknown, it deserves qualitative and quantitative assessment.

## Conclusion

A gesture and attitude of social responsibility promoted by a global icon, capable of positive influence in behavioral change, that will save lives.

## Disclosure of interest

None declared.

